# Supplementary material for: Rerupture outcome of conservative versus open repair versus minimally invasive repair of acute Achilles tendon ruptures: A systematic review and meta-analysis
Source: PLoS One. 2023 May 2;18(5):e0285046. doi: 10.1371/journal.pone.0285046 (PMC10153690; doi:10.1371/journal.pone.0285046)
Supplement: S1 Table — (DOCX) [file pone.0285046.s001.docx]

**S1 Table. Search strategy**

| **MEDLINE(R)** | | |
| --- | --- | --- |
|  | exp achilles tendon/ | 9199 |
|  | achill*.ab. | 10575 |
|  | tendoachill*.ab. | 68 |
|  | calcaneal*.ab. | 4651 |
|  | calcanean*.ab. | 32 |
|  | calcaneus.ab. | 4510 |
|  | or/1-6 | 20638 |
|  | exp rupture/ | 51779 |
|  | Tendon Injuries.ab. | 1455 |
|  | ruptu*.ab. | 97327 |
|  | injur*.ab. | 711635 |
|  | lesion*.ab. | 761973 |
|  | tear*.ab. | 45633 |
|  | or/8-13 | 1556928 |
|  | exp surgical procedures, operative/ | 3451433 |
|  | exp orthopedics/ | 23172 |
|  | surg*.ab. | 1597429 |
|  | operat*.ab. | 989688 |
|  | orthop*.ab. | 82908 |
|  | kessler.ab. | 1874 |
|  | bunnell.ab. | 293 |
|  | krackow.ab. | 156 |
|  | achillon.ab. | 38 |
|  | tenolig.ab. | 7 |
|  | dresden.ab. | 895 |
|  | percuta*.ab. | 122218 |
|  | or/15-26 | 4645936 |
|  | exp Conservative Treatment/ | 4686 |
|  | conservative.ab. | 90246 |
|  | conventional.ab. | 444358 |
|  | non-operative.ab. | 4316 |
|  | non operative.ab. | 4316 |
|  | nonoperative.ab. | 9219 |
|  | non-surgical.ab. | 10177 |
|  | non surgical.ab. | 10177 |
|  | nonsurgical.ab. | 13159 |
|  | cast*.ab. | 83349 |
|  | brace*.ab. | 6018 |
|  | splint*.ab. | 10692 |
|  | boot*.ab. | 19683 |
|  | bandage*.ab. | 4004 |
|  | tape.ab. | 13491 |
|  | taping.ab. | 1657 |
|  | or/28-43 | 694197 |
|  | 7 and 14 and 27 and 44 | 914 |
|  | randomized controlled trial.pt. | 574731 |
|  | controlled clinical trial.pt. | 94968 |
|  | randomized.ab. | 503802 |
|  | randomised.ab. | 102994 |
|  | placebo.tw. | 217387 |
|  | clinical trials as topic.sh. | 200270 |
|  | randomly.ab. | 332374 |
|  | trial.ti. | 237010 |
|  | or/46-53 | 1368192 |
|  | exp animals/ not humans.sh. | 5036968 |
|  | 54 not 55 | 1249303 |
|  | 45 and 56 | 167 |
| **EMBASE** | | |
|  | 'achilles tendon rupture'/de | 3,013 |
|  | 'achilles tendon'/exp | 11617 |
|  | 'achilles tendon':ab,ti | 10691 |
|  | 'achill*':ab,ti | 19,559 |
|  | 'tendoachill*':ab,ti | 280 |
|  | 'calcanean*':ab,ti | 67 |
|  | 'calcaneus':ab,ti | 7385 |
|  | #1 OR #2 OR #3 OR #4 OR #5 OR #6 OR #7 | 29307 |
|  | 'rupture'/de | 39622 |
|  | 'tendon injury'/de | 10156 |
|  | 'injur*':ab,ti | 1215225 |
|  | 'ruptu*':ab,ti | 179641 |
|  | 'lesion*':ab,ti | 1301757 |
|  | 'tear*':ab,ti | 76139 |
|  | #9 OR #10 OR #11 OR #12 OR #13 OR #14 | 2637839 |
|  | 'surgery'/de | 691401 |
|  | 'orthopedic surgery'/de | 38464 |
|  | 'surg*':ab,ti | 3040262 |
|  | 'operat*':ab,ti | 1719923 |
|  | 'orthop*':ab,ti | 162761 |
|  | 'kessler':ab,ti | 2941 |
|  | 'bunnell':ab,ti | 543 |
|  | 'krackow':ab,ti | 232 |
|  | 'achillon':ab,ti | 53 |
|  | 'tenolig':ab,ti | 16 |
|  | 'dresden':ab,ti | 2343 |
|  | 'percuta*':ab,ti | 249016 |
|  | #16 OR #17 OR #18 OR #19 OR #20 OR #21 OR #22 OR #23 OR #24 OR #25 OR #26 OR #27 | 4470276 |
|  | 'conservative treatment'/de | 95787 |
|  | 'conservative':ab,ti | 159811 |
|  | 'conventional':ab,ti | 724541 |
|  | 'non-operative':ab,ti | 8970 |
|  | 'non operative':ab,ti | 8948 |
|  | 'nonoperative':ab,ti | 21898 |
|  | 'non-surgical':ab,ti | 21981 |
|  | 'non surgical':ab,ti | 21955 |
|  | 'nonsurgical':ab,ti | 40388 |
|  | 'cast*':ab,ti | 142973 |
|  | 'brace*':ab,ti | 10782 |
|  | 'splint*':ab,ti | 18715 |
|  | 'boot*':ab,ti | 33073 |
|  | 'bandage*':ab,ti | 7633 |
|  | 'tape':ab,ti | 24651 |
|  | 'taping':ab,ti | 3026 |
|  | #29 OR #30 OR #31 OR #32 OR #33 OR #34 OR #35 OR #36 OR #37 OR #38 OR #39 OR #40 OR #41 OR #42 OR #43 OR #44 | 1196294 |
|  | 'randomized controlled trial' | 974091 |
|  | 'controlled clinical trial' | 465421 |
|  | randomized:ab | 828493 |
|  | randomised:ab | 164335 |
|  | Placebo | 509201 |
|  | 'clinical trial (topic)' | 194795 |
|  | randomly:ab | 515706 |
|  | trial:ab | 897564 |
|  | #46 OR #47 OR #48 OR #49 OR #50 OR #51 OR #52 OR #53 | 2544638 |
|  | 'human'/exp | 25147341 |
|  | #8 AND #15 AND #28 AND #45 AND #54 AND #55 | 225 |
| **COCHRANE CENTRAL** | | |
|  | Achilles | 1388 |
|  | rupture | 7107 |
|  | #1 and #2 | 345 |
