# Supplementary material for: Rerupture outcome of conservative versus open repair versus minimally invasive repair of acute Achilles tendon ruptures: A systematic review and meta-analysis
Source: PLoS One. 2023 May 2;18(5):e0285046. doi: 10.1371/journal.pone.0285046 (PMC10153690; doi:10.1371/journal.pone.0285046)
Supplement: S2 Table — T1: treatment 1; T2: treatment 2; I2.pair: I-square of pair-wise meta-analysis; I2.cons: I-square of network meta-analysis; Incons.p: inconsistency p-values for pair-wise and network meta-analysis. (DOCX) [file pone.0285046.s002.docx]

**S2 Table. Analysis of heterogeneity for rerupture rate**

| Per-comparison I-squared: | | | | | |
| --- | --- | --- | --- | --- | --- |
|  | T1 | T2 | I^2^ .pair | I^2^ .cons | Incons .p |
| 1 | conservative | Open repair | 0 | 0 | 0.83 |
| 2 | conservative | minimally invasive surgery | 0 | 0 | 0.86 |
| 3 | Open repair | minimally invasive surgery | 0 | 0 | 0.96 |
| Global I-squared: | | | | | |
|  | I^2^ .pair | I^2^ .cons | | |  |
| 1 | 0 | 0 |  |  |  |

T1: treatment 1; T2: treatment 2; I^2^ .pair: I-square of pair-wise meta-analysis; I^2^ .cons: I-square of network meta-analysis; Incons .p: inconsistency p-values for pair-wise and network meta-analysis;
