# Supplementary figures and images for: Rerupture outcome of conservative versus open repair versus minimally invasive repair of acute Achilles tendon ruptures: A systematic review and meta-analysis
Source: PLoS One. 2023 May 2;18(5):e0285046. doi: 10.1371/journal.pone.0285046 (PMC10153690; doi:10.1371/journal.pone.0285046)

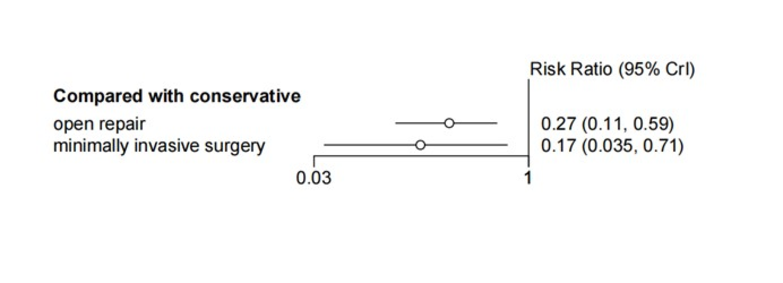

Supplement: S1 Fig — (TIF) [file pone.0285046.s003.tif]

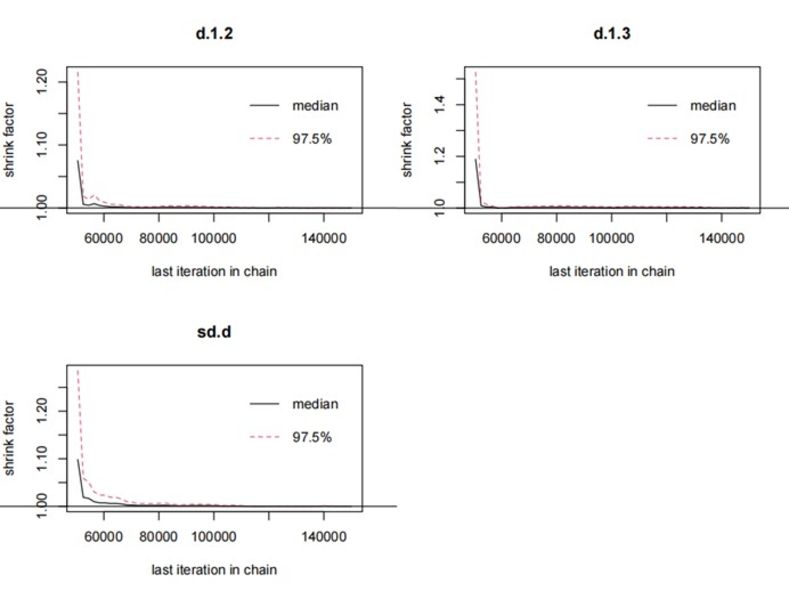

Supplement: S2 Fig — (TIF) [file pone.0285046.s004.tif]

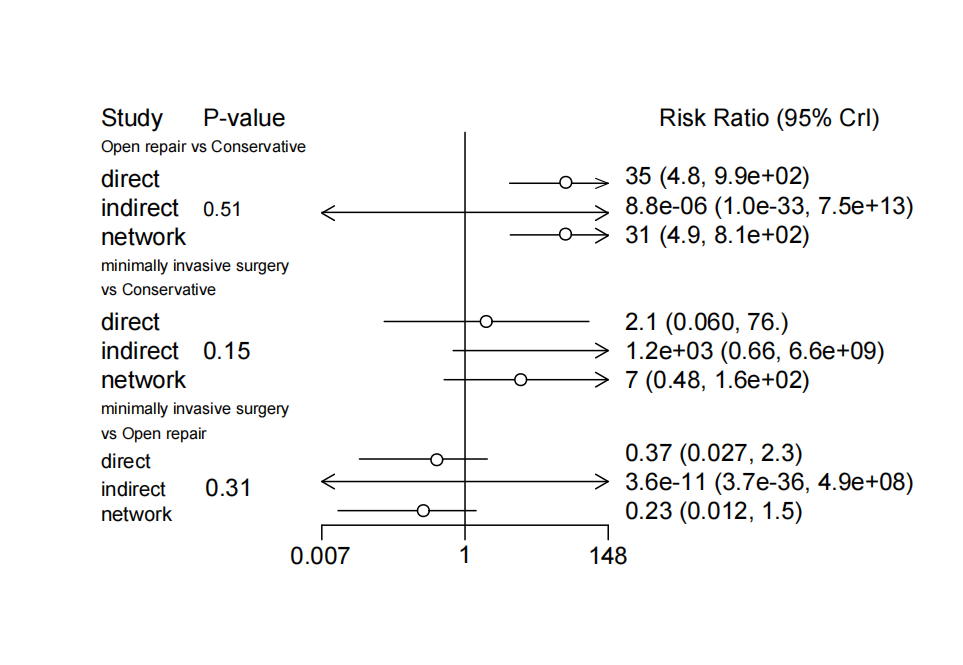

Supplement: S3 Fig — (TIF) [file pone.0285046.s005.tif]

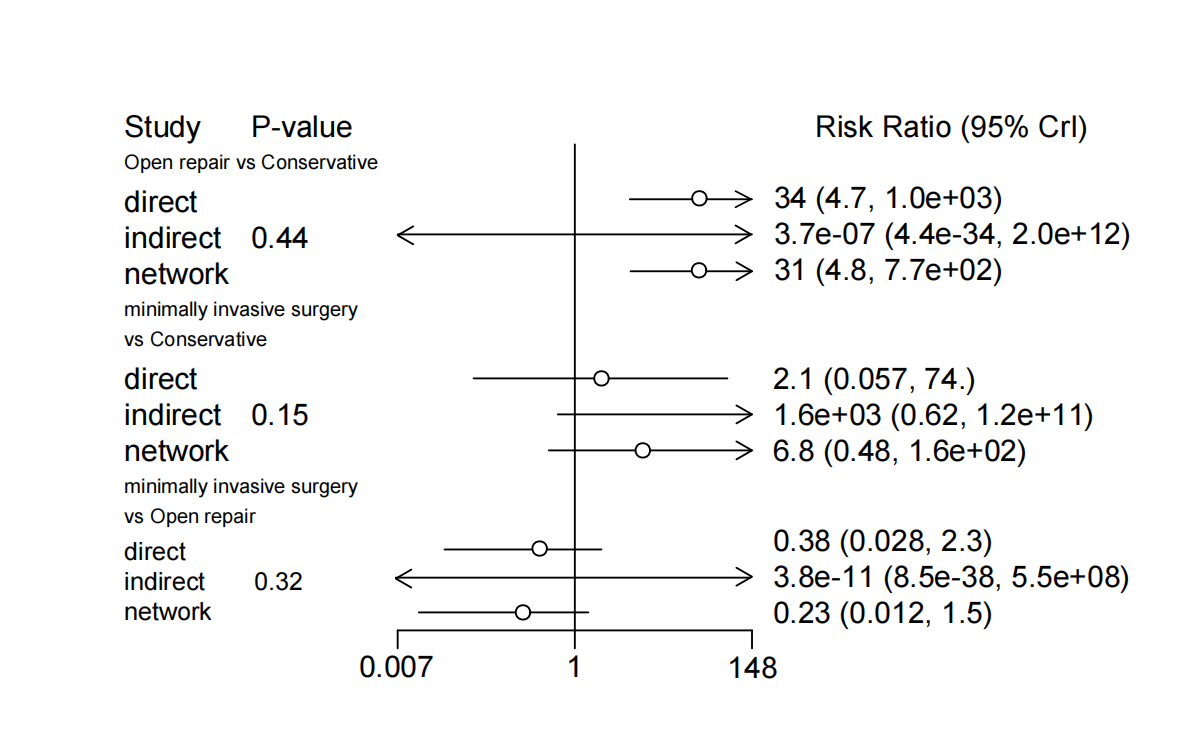

Supplement: S4 Fig — (TIF) [file pone.0285046.s006.tif]

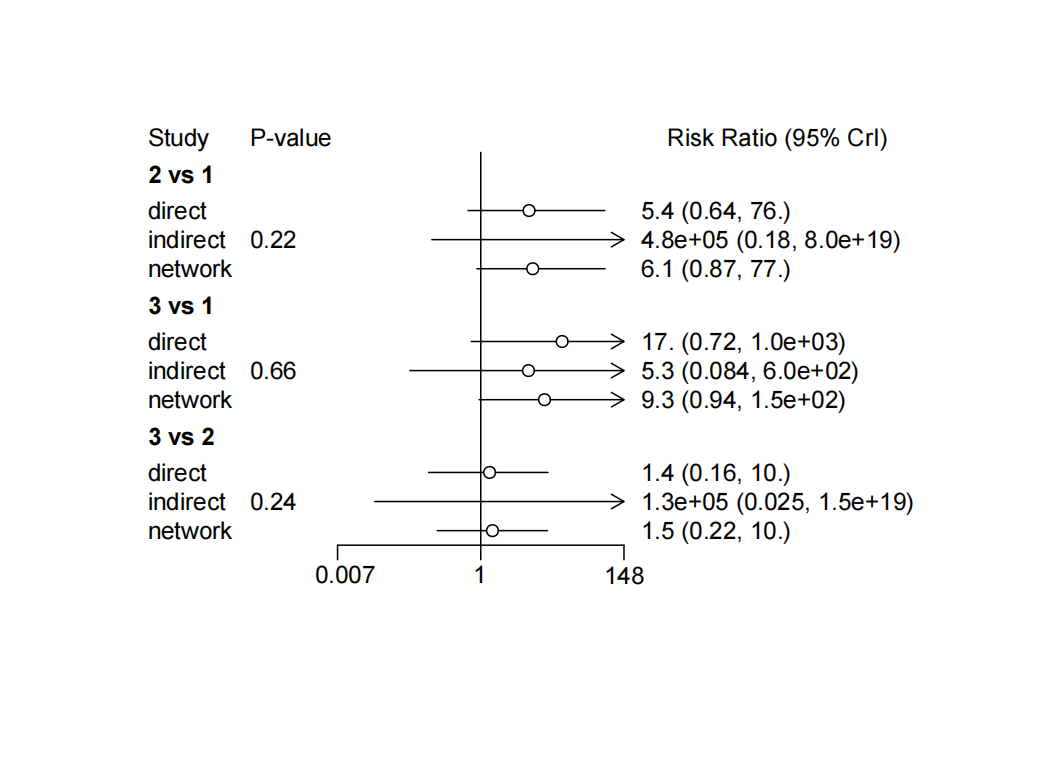

Supplement: S5 Fig — (TIF) [file pone.0285046.s007.tif]

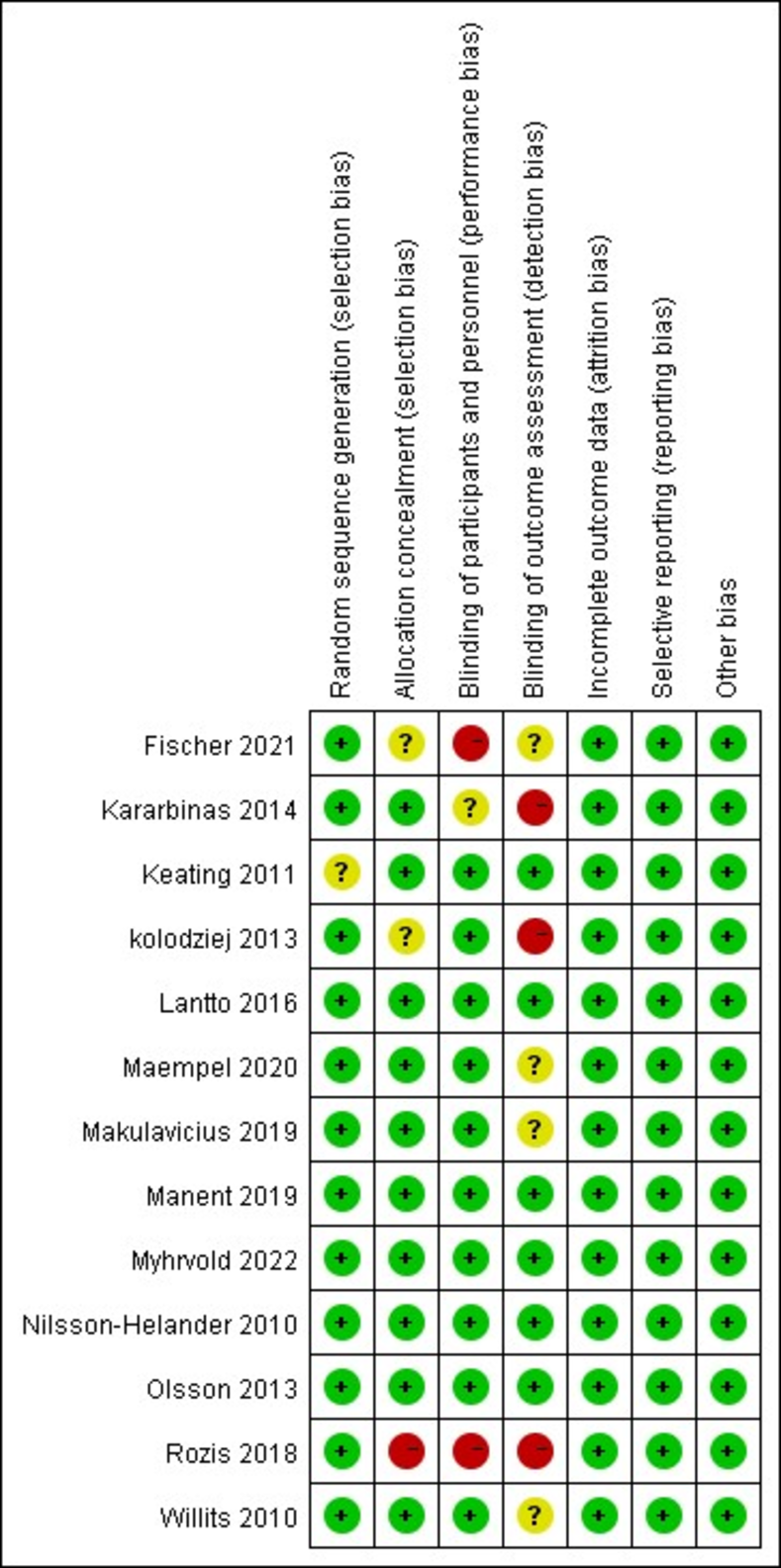

Supplement: S6 Fig — (TIF) [file pone.0285046.s008.tif]

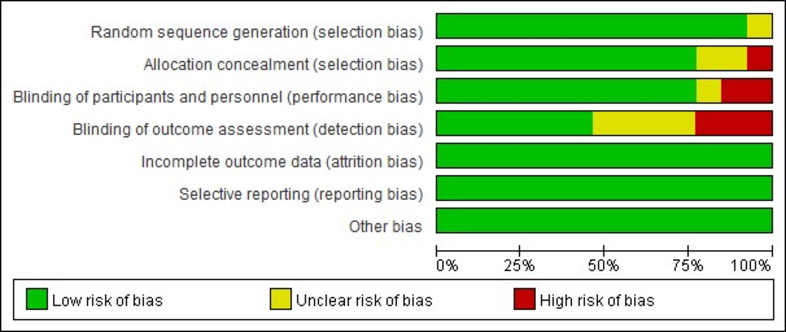

Supplement: S7 Fig — (TIF) [file pone.0285046.s009.tif]
